# *In vitro* study: HIF-1α-dependent glycolysis enhances NETosis in hypoxic conditions

**DOI:** 10.3389/fimmu.2025.1583587

**Published:** 2025-04-28

**Authors:** Yi Ye, Yanjun Wang, Qiying Xu, Juanli Liu, Ziqi Yang, Tana Wuren, Ri-Li Ge

**Affiliations:** ^1^ Research Center for High Altitude Medicine, Qinghai University, Xining, China; ^2^ High-Altitude Medicine Key Laboratory of the Ministry of Education, Xining, China; ^3^ Qinghai Provincial Key Laboratory for Application of High-Altitude Medicine, Xining, China; ^4^ Department of Geriatrics, Qinghai University Affiliated Hospital, Xining, China; ^5^ Department of Gynecology, Qinghai University Affiliated Hospital, Xining, China; ^6^ Department of Critical Care Medicine, Qinghai Provincial People's Hospital, Xining, China; ^7^ Medical College of Qinghai University, Xining, China

**Keywords:** HIF-1α, glycolysis, neutrophil extracellular traps, hypoxia, neutrophils

## Abstract

**Background:**

Hypoxia plays a pivotal role in modulating immune responses, especially in neutrophils, which are essential components of the innate immune system. Hypoxia-inducible factor (HIF)-1α, a key transcription factor in hypoxic adaptation, regulates cellular metabolism and inflammatory responses. However, the impact of HIF-1α-dependent glycolysis on the formation of neutrophil extracellular traps (known as NETosis) under hypoxic conditions remains unclear.

**Methods:**

We employed two established neutrophil models, neutrophils isolated from human whole blood and DMSO-induced dHL-60 cells, to explore the role of HIF-1α in regulating glycolysis and its influence on NETosis under hypoxic conditions. We utilized western blotting, immunofluorescence staining, ELISA, and flow cytometry to evaluate the expression of key glycolytic enzymes and NETosis markers under hypoxia. Additionally, the effects of inhibiting HIF-1α with LW6 and blocking the glycolytic pathway with Bay-876 were investigated.

**Results:**

HIF-1α-dependent glycolysis, through the upregulation of key glycolytic enzymes, significantly enhances NETosis under hypoxic conditions. Pharmacological inhibition of HIF-1α with LW6 and glycolytic blockade with Bay-876 markedly reduced NETosis, underscoring the crucial role of metabolic reprogramming in neutrophil function during hypoxia.

**Conclusion:**

This study provides novel insights into the interplay between metabolic reprogramming and NETosis in response to hypoxic stress. We identify HIF-1α-dependent glycolysis as a key driver of NETs formation, advancing our understanding of the mechanisms underlying hypoxia-related inflammatory diseases. These findings also suggest that targeting metabolic pathways may offer potential therapeutic strategies for modulating immune responses in hypoxia-associated disorders.

## 1 Introduction

Hypoxia refers to a state where oxygen concentration is below 21% or when tissue oxygen supply is insufficient to meet cellular demands (1). It can be classified into macroscopic and microscopic types. Macroscopic hypoxia occurs in large-scale, external low-oxygen environments, such as high-altitude areas, where reduced oxygen availability triggers systemic inflammatory responses in both acute and chronic mountain sickness (2). Microscopic hypoxia occurs at the cellular or tissue level and can be further divided into physiological and pathological hypoxia. Physiological hypoxic regions include the intestinal mucosa, bone marrow, placenta, retina, and lymph nodes (3), while pathological hypoxia is commonly found in tumors (4), inflamed tissues, and ischemic regions (5). In inflamed tissues, hypoxia promotes immune cell recruitment and the release of inflammatory mediators (6), triggering microvascular inflammation, which increases vascular permeability and enhances leukocyte-endothelial cell adhesion and migration (7), amplifying the inflammatory response while facilitating pathogen clearance. In turn, inflammation exacerbates hypoxia by increasing oxygen consumption, reducing oxygen supply, impairing mitochondrial function, and inducing oxidative stress, leading to an imbalance in oxygen supply and demand (8). In this interplay, Hypoxia-inducible factor (HIF)-1α acts as a key regulator, driving aberrant gene transcription and further intensifying inflammation (5). Thus, the inflammatory microenvironment and oxygen metabolism are intricately linked (9), with HIF-1α as the central mediator (10).

*In vivo* models show that the increased survival of neutrophils under hypoxic conditions is primarily mediated by HIF-1α stabilization (11). Hypoxia induced in healthy volunteers in a chamber simulating conditions at 5000 meters increases HIF-1α protein expression in neutrophils, activating the NF-κB pathway and promoting neutrophil survival (12). In a chronic inflammation model in zebrafish, hypoxia and HIF-1α were shown to delay neutrophil apoptosis and reverse neutrophil migration from the wound, thus prolonging inflammation and exhibiting pro-inflammatory characteristics (13). Moreover, HIF-1α has been shown to significantly affect various neutrophil functions under hypoxic conditions, including phagocytosis, cell surface marker expression, cytokine secretion, chemokine receptor levels, adhesion, and migration (14), thereby enhancing their overall antimicrobial capacity (15–17).

Neutrophils heavily rely on anaerobic glycolysis to generate ATP (18, 19), enabling their migration to inflammation sites characterized by low oxygen and glucose levels and high levels of reduced metabolites (20). Recent studies have identified HIF-1α as a key regulator that controls neutrophil-mediated inflammation by driving the metabolic shift toward glycolysis (14, 21). In HIF-1α conditional knockout mice, the loss of HIF-1α depletes the ATP pool in neutrophils, severely impairing neutrophil aggregation, motility, bacterial killing, and invasion (22). This study establishes HIF-1α as a crucial metabolic sensor in neutrophils and provides direct evidence of the link between HIF-1α-mediated oxygen sensing mechanisms and metabolic reprogramming in neutrophils. Under hypoxic conditions, enhanced glycolysis within neutrophils promotes the synthesis of mitochondrial reactive oxygen species (ROS), stabilizes HIF-1α, and supports neutrophil survival (23), thereby reinforcing their immune functions.

In addition to phagocytosis, intracellular degradation, and the rapid release of granules that perform diverse functions to combat invading pathogens, neutrophils can also capture and eliminate pathogens by releasing extracellular structures known as NETs (24, 25). Neutrophils form NETs in response to a variety of stimuli (26) such as pathogens, inflammatory factors (27) (IL-8, IL-1β, IFNα), ROS (28), chemicals (e.g., sodium arsenite, animal toxins, ethyl myristoylphorbol (29) (Phorbol 12-myristate 13-acetate [PMA] etc.) and platelet-activating factor activated and formed in response to various stimuli (30).

NETosis is a dynamic process involving several stages, beginning with the increasing permeability of the nuclear membrane and chromatin decondensation. During this process, the contents of the nucleus mix with cytoplasmic material and granule proteins. In the final stage, both nuclear and granular components are released into the extracellular space (31). The DNA released forms a fibrous scaffold, which supports the antimicrobial activity of histones, proteases such as myeloperoxidase (MPO) and neutrophil elastase (NE), and antimicrobial peptides (AMPs) like cathelicidins (25). These components collectively contribute to the immune defense against pathogens. Although the importance of HIF-1α in the traditional immune functions of neutrophils has been well established, its role in regulating NETosis under hypoxic conditions remains to be fully elucidated.

Given the central role of HIF-1α in the response to hypoxia and the known impact of glycolytic reprogramming on neutrophil function, elucidating the relationship between HIF-1α-dependent glycolysis and NETosis under hypoxic conditions is critical for gaining insight into immune regulation in inflammatory diseases. To address this gap, our study utilized two well-established neutrophil models: neutrophils isolated from human whole blood and DMSO-induced dHL-60 cells, to investigate the role of HIF-1α in regulating glycolysis and its impact on NETosis under hypoxic conditions. Using Western blotting, immunofluorescence staining, and flow cytometry, we assessed the expression of key glycolytic enzymes and NETosis markers under hypoxia. Further, we evaluated the effects of inhibiting HIF-1α with LW6 and blocking the glycolytic pathway with Bay-876. Our findings indicate that HIF-1α-driven glycolysis significantly enhances NETosis in hypoxic environments, providing new insights into how metabolic reprogramming during hypoxic stress influences innate immune responses. Understanding the molecular mechanisms connecting HIF-1α, glycolysis, and NETosis under hypoxia not only sheds light on the pathogenesis of diseases characterized by hypoxia and inflammation but also suggests potential therapeutic strategies for modulating immune responses through metabolic pathway regulation.

## 2 Materials and Methods

### 2.1 Isolation of neutrophils from whole undiluted human blood

Whole undiluted human blood (n=6) was obtained from healthy donors. Experimental procedures using human whole blood were approved by the Affiliated Hospital of Qinghai University (Approval Number: SL-2020091). Whole blood was collected into an EDTA tube and processed within 2 to 4 h after collection. It was then carefully layered onto 4 mL of Polymorphprep gradient medium (Cosmo Bio USA, AXS-1114683) at a 1:2 (v/v) ratio, ensuring that no mixing occurred. The mixture was centrifuged at room temperature for 30 min at 500 × g to achieve gradient separation, as illustrated in **Supplementary Figure S1**. Cell-containing rings indicative of neutrophil rings were transferred into a new centrifugal tube.

### 2.2 Evaluation of viability and purity of the isolated neutrophils

Single-cell suspensions were prepared from the neutrophil isolates, followed by red blood cell lysis (420301, BioLegend, San Diego, CA, USA). Neutrophils were identified based on side and forward scatter profiles and then analyzed for the presence of the APC-conjugated anti-human CD15 antibody (BioLegend, 301904), a specific marker for neutrophil surface expression (32). See **Supplementary Figures S2** and **S3** for an overview of the gating strategy and the results for a representative cohort. The results showed that the isolated cells were of high quality, met the experimental requirements, and could be used for subsequent experimental studies.

### 2.3 Differentiation and characterization of HL-60 cells

The HL-60 cell line was obtained from Wuhan Pricella Biotechnology Co., Ltd. HL-60 cells were cultured in complete IMDM (CM-0110, Pricella) at 37°C in a 5.0% CO_2_ incubator. Cells were passaged every 3 days to maintain a density of 3×10^5^ to 5×10^5^ cells/mL.HL-60 cells were induced to differentiate using 1.3% dimethyl sulfoxide (DMSO) and incubated undisturbed for 6 days at 37°C in 5.0% CO_2_.

To validate the effectiveness of this differentiation method in constructing an *in vitro* neutrophil model, Giemsa staining (**Supplementary Figure S4**) was first performed to observe morphological changes in HL-60 cells after differentiation. The presence of segmented nuclei and characteristic neutrophil-like morphology confirmed successful differentiation.

Subsequently, differentiation efficiency was further evaluated by flow cytometry, assessing CD11b expression as a neutrophil differentiation marker. Cells were stained with a FITC-conjugated anti-human CD11b antibody (982614, BioLegend), and dead cells were excluded using the Zombie Aqua Fixable Viability Kit (423101, BioLegend). Flow cytometry was performed using a BD FACSAria III flow cytometer (BD Biosciences), and data were analyzed using FlowJo software. The gating strategy, CD11b expression levels, and cell viability analysis results are shown in **Supplementary Figures S5–S7**.

### 2.4 NETs induction *in vitro* by PMA

Phorbol 12-myristate 13-acetate (PMA) is a potent ROS inducer activated by protein kinase C (PKC) and, therefore, can be used as a positive control for the induction of NETosis (33). The neutrophils were resuspended in RPMI 1640 (11835030, Gibco, Thermo Fisher Scientific, USA) and plated on poly-l-lysine (J24001, Shanghai jing An biological technology co., LTD) coated coverslips at a density of 2 × 10^5^ cells/well in 24-well plates (702011, Wuxi NEST Biotechnology Co., Ltd). After preincubation for 30 min at 37°C, 5.0% CO_2_, they were stimulated with 100 nM PMA (P1585, Sigma-Aldrich, St. Louis, MO) and incubated for 4 h at 37°C, 5.0% CO_2_.

### 2.5 Hypoxic stimulation and inhibition of HIF-1α and GLUT1

A poly-L-lysine-coated coverslip was placed into each well of a 24-well cell culture plate. Differentiated HL-60 cells (dHL-60) were seeded at a density of 2 × 10^5^ cells per well in 500 μL RPMI medium and incubated for 30 min in a CO_2_ incubator at 37°C. For hypoxic stimulation, cells were exposed to either normoxic conditions (mixture of 95% ambient air and 5% CO_2_) or hypoxic conditions (1.0% O_2_, 5.0% CO_2_, and 94.0% N_2_) at 37°C. Hypoxic conditions were maintained using a hypoxic workstation (Defendor HW 2000, Hariolab, China) with a gas flow rate of 20 L/min. The workstation was purged with gas, sealed, and placed in a conventional incubator at 37°C for 4 h. To inhibit HIF-1α expression, 10 μM LW6 (HY-13671, MedChemExpress, USA) was added to the dHL-60 cells following the initial 30-minute incubation. Bay-876 (HY-100017, MedChemExpress, USA) was used under the same conditions to inhibit the glycolytic pathway. The negative control group was treated under the same conditions but without the inhibitors.

### 2.6 NETs detection by immunofluorescence

Neutrophils were seeded onto coverslips in a 24-well plate and stimulated under normoxic, hypoxic, or PMA conditions as positive controls for NETs release. The plate was then transferred to a chemical fume hood, and 500 μL of 4% PFA was added to fix the cells for at least 20 min at room temperature. After fixing, cells were washed three times with Hank’s Balanced Salt Solution (HBSS) for 5 min each. The blocking step was performed by adding 500 μL of blocking buffer (3% BSA in PBS) and incubating for 30 min at room temperature. Next, a 10 nM dilution of SYTOX Green staining solution (S7020, Thermo Fisher Scientific) was applied to cover the cells, and the cells were incubated for 15 min in the dark. After incubation, the staining solution was removed, and the cells were washed three times with HBSS and twice with distilled water. The coverslips were dried on a paper towel before 5 μL of antifade mounting solution with DAPI (C0065, Solarbio Life Science, China) was added. The coverslip was carefully placed onto a clean, degreased microscope slide with the sample facing down. The slide was left for 30 min to allow any excess fluid to evaporate. Imaging was performed using the Nikon DS-U3 Microscope System.

### 2.7 Measurement of NETosis by flow cytometry

Groups of dHL-60 cells receiving hypoxia and PMA stimulation were suspended in PBS with Zombie Aqua Fixable Viability Kit to exclude dead cells. Without a permeabilization step, cells were incubated sequentially with the primary anti-Histone H3 (citrulline R2+R8+R17) antibody (ab5103, 1:100; Abcam, Cambridge, United States), Alexa Fluor700-conjugated secondary antibody (A-21038, 1:100; Thermo Scientific), FITC-conjugated anti-myeloperoxidase antibody (ab11729, 1:100; Abcam) and APC-conjugated anti-human CD11b antibody(301310, BioLegend). Each incubation was followed by a wash with 2% BSA in PBS and centrifugation at 2000 rpm at 4°C for 5 min. Samples were then resuspended in PBS and analyzed by BD FACSAria III flow cytometer (BD Biosciences). Files were analyzed in FlowJo. An overview of the gating strategy is shown in **Supplementary Figure S8**.

### 2.8 ELISA detection of plasma neutrophil elastase

Plasma NE levels were detected using the Human NE/ELA2 (Neutrophil Elastase/Elastase-2) ELISA Kit (E-EL-H1946, Elabscience Biotechnology Co., Ltd. China). Plasma samples were processed and analyzed according to the manufacturer’s instructions. Briefly, the plasma samples were added to the wells of the pre-coated ELISA plate, followed by incubation with the corresponding detection antibody. After washing, the TMB substrate solution was added, and the reaction was stopped, yielding a colorimetric signal proportional to the NE concentration. Absorbance was measured at 450 nm using an automated plate reader. All procedures were carried out in accordance with the manufacturer’s protocol.

### 2.9 Western blot

Total protein was lysed using RIPA Buffer (R0010, Solarbio Life Science). Equal amounts of protein (25–40 μg per lane) were separated by SDS-PAGE and transferred to PVDF membranes (Millipore, Burlington, MA, USA). The membranes were blocked with 5% non-fat dry milk in TBST for 1 h, followed by overnight incubation at 4°C with the appropriate primary antibodies: HIF-1α rabbit monoclonal antibody (D2U3T,1:1000; CST), Anti-Histone H3 (citrulline R2 + R8 + R17) antibody (ab5103, 1:1000; Abcam), Histone H3 Monoclonal antibody(68345-1-Ig, 1:5000; Proteintech, China), Anti-Glucose Transporter GLUT1 antibody (ab115730, 1:100 000; Abcam), Anti-Hexokinase II antibody (ab209847, 1:1000; Abcam), Anti-PFKFB3 antibody (ab181861, 1:1000; Abcam), GAPDH Monoclonal antibody (60004-1-Ig, 1:50 000; Proteintech), ENO1 Polyclonal antibody (11204-1-AP, 1:2000; Proteintech), Anti-PKM antibody (ab150377, 1:10 000; Abcam), Anti-Lactate Dehydrogenase antibody (ab52488,1:5000; Abcam) and beta-actin rabbit polyclonal antibody (20536-1-AP, 1:5000; Proteintech). The membranes were subsequently incubated with an appropriate HRP-conjugated secondary antibody for 1 hour, followed by detection using the SuperKine ultra-sensitive ECL substrate (BMU102-CN/BMU103-CN; Abbkine, Wuhan, China). The signals were then quantified using ImageJ software (NIH, Bethesda, MD, United States).

## 3 Statistical analysis

Statistical analyses were conducted using GraphPad Prism 10 (GraphPad Software, San Diego, CA, USA). The normality of the data was assessed using the Shapiro-Wilk test, with a significance level of α = 0.05. For comparisons between multiple groups, ANOVA followed by Dunnett’s Multiple Comparison was used. All results are expressed as means ± standard error of the mean (SEM). Statistical significance was determined with the following thresholds: *p<0.05, **p<0.01, ***p<0.001, ****p<0.0001, ns = not significant.

## 4 Results

### 4.1 Hypoxia promotes NETosis

Hypoxia is a prominent feature of both physiological and pathological immune microenvironments (5). At sites of inflammation or the central necrotic regions of solid tumors, nearly 95% of effector cells of the innate immune system, including neutrophils, are recruited to these areas rather than residing there (14). Consequently, neutrophils must migrate against oxygen gradients to reach these relevant inflammatory sites (34). Previous studies have demonstrated that hypoxia not only directly regulates neutrophil survival under low-oxygen conditions but also enhances their anti-apoptotic, degranulation, and metabolic responses.

To further investigate the effect of hypoxia on NETosis, freshly isolated peripheral blood neutrophils were incubated under normoxic (21%) and hypoxic (1%) conditions. NETosis was qualitatively and semi-quantitatively assessed using two fluorescent dyes with different DNA-binding properties: SYTOX Green and DAPI (35). PMA (100 nM) was used as a positive control.

**Figure 1:**
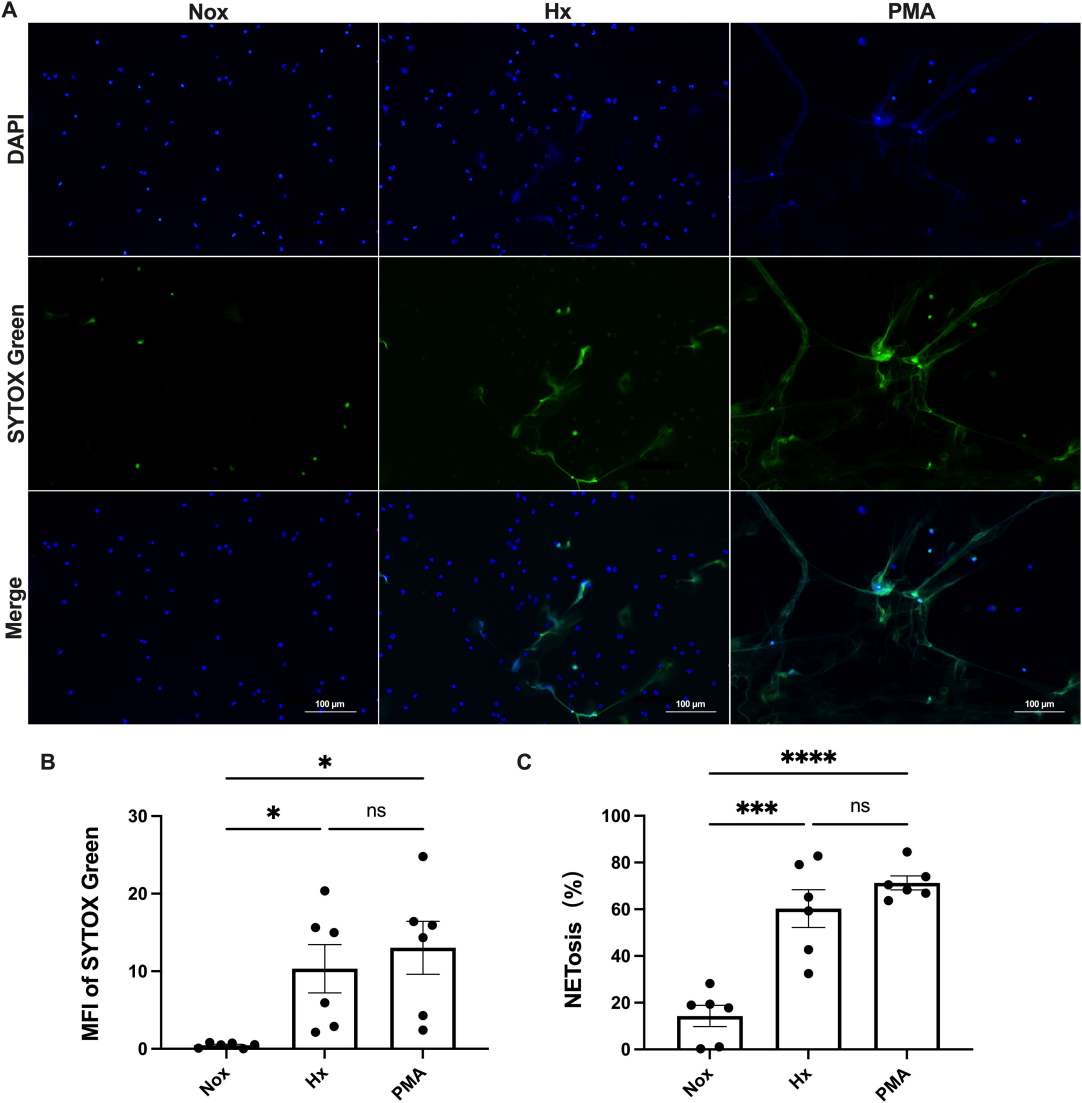
Hypoxia induces NET formation in human blood-derived neutrophils. Neutrophils were exposed to either normoxic (Nox) or hypoxic (Hx) conditions for 4 h or treated with 100 nM PMA as a positive control. NET formation was visualized using SYTOX Green, a DNA-binding dye that is impermeable to cells (green), in combination with DAPI to stain the nuclei (blue). The images were captured at 40× magnification; scale bar = 100 μm. **(A)** Representative fluorescence micrographs showing NET release from neutrophils. **(B)** Quantification of the mean fluorescence intensity (MFI) of each group of SYTOX Green. **(C)** Percentage of NETosis. All conditions were compared to untreated controls (normoxia), sample sizes (n)=6. *p < 0.05, ***p < 0.00, ****p < 0.0001, ns, not significant.

Immunofluorescence analysis revealed that hypoxia significantly promoted NETosis in peripheral blood neutrophils (**Figure 1A**). Compared to the normoxic group, neutrophils in the hypoxic group exhibited diffuse nuclear staining and a marked enhancement in SYTOX Green fluorescence, indicating increased extracellular DNA release. Semi-quantitative analysis (**Figures 1B, C**) further confirmed that the average fluorescence intensity of SYTOX Green and the percentage of NETs generated in the hypoxia group were significantly higher than those in the normoxia group (p < 0.05; p < 0.001), suggesting that hypoxia effectively induces NETs formation. Notably, no significant differences were observed between the hypoxia group and the PMA-positive control group in these two parameters (p > 0.05), indicating that hypoxic stimulation promoted NETs generation at a level comparable to that induced by PMA.

### 4.2 Time-dependent and HIF-1α sensitive increases in NETosis under hypoxia exposure

The duration of hypoxic exposure often triggers distinct cellular and tissue responses (36), with HIF-1α being recognized as the central mediator of the hypoxic response (37). After confirming that hypoxia induces NETosis in neutrophils isolated from healthy donor whole blood, we further investigated the time-dependent effects of hypoxia on neutrophils and their participation in NETosis at different time points of exposure.

**Figure 2:**
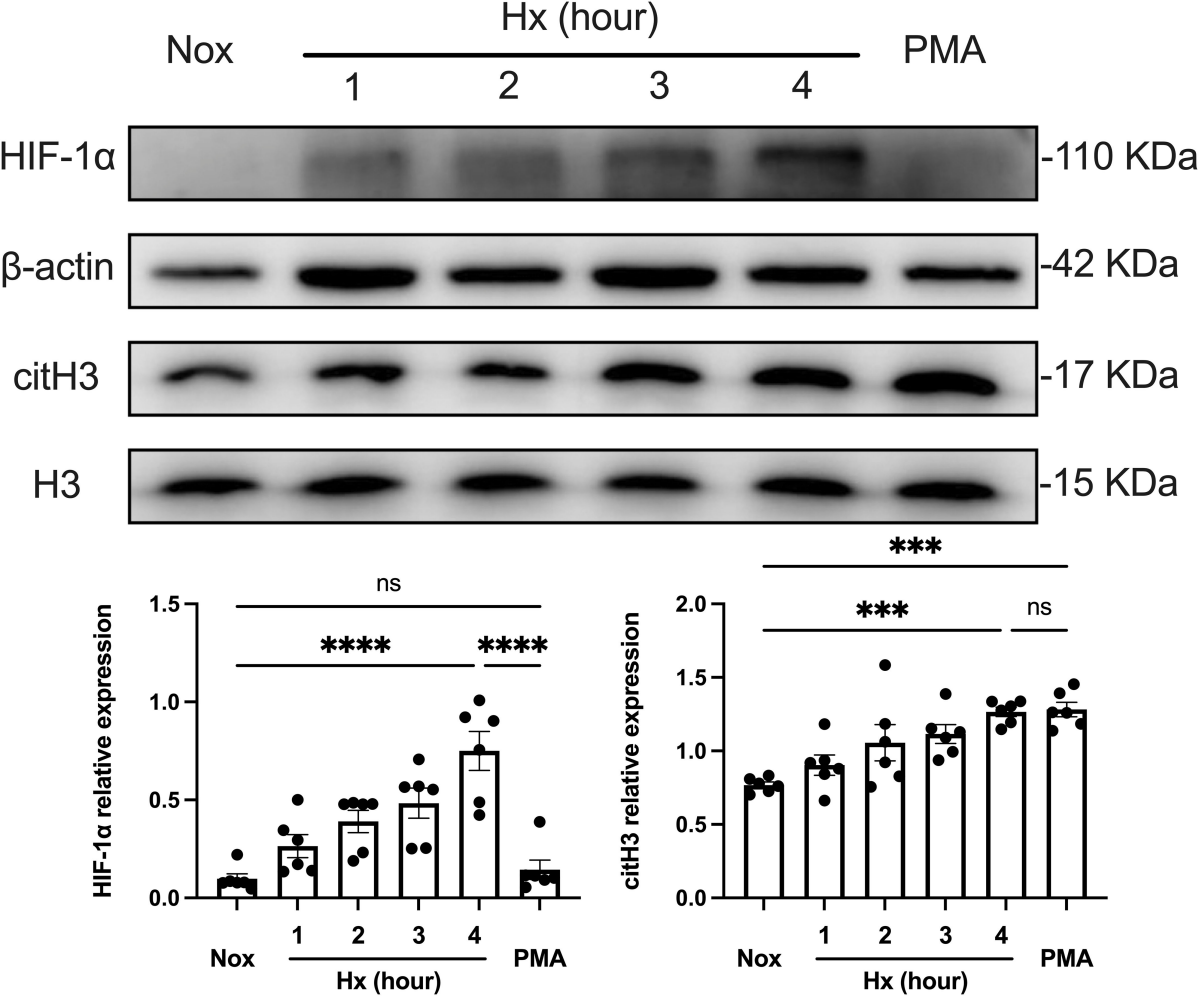
Time-dependent effect of hypoxia on NETosis formation. The upper part shows the expression of HIF-1α and citH3 in dHL−60 cells incubated under hypoxic conditions for various times (0–4 h) with or without PMA, assessed by Western blot analysis, with β-actin and H3 as loading controls. The lower part shows the quantification of HIF-1α and citH3 protein levels normalized to β-actin or H3, respectively. n = 6. ***p < 0.001, ****p < 0.0001, ns, not significant.

Given the research limitations of primary neutrophils with short lifespans, variable donors, and low transcriptional activity (38), we referred to the method of Bhakta et al. (39, 40), where human leukemia (HL-60) cells were induced with 1.3% DMSO for 6 consecutive days to induce differentiation into neutrophil-like (dHL-60) cells to construct a classical neutrophil model for *in vitro* study. After confirming cell differentiation using Giemsa staining as well as flow cytometry, dHL-60 cells were treated under hypoxic conditions for different times (0–4 h), and PMA was used as a positive control. The induction of NETosis in dHL-60 cells by hypoxia was further confirmed by analyzing the expression levels of intracellular HIF-1α and citrullinated histone H3 (citH3) by western blot (**Figure 2**). HIF-1α protein expression was strongly increased by hypoxia, and this increase persisted when the exposure to hypoxia was extended. This result suggests that the stability and expression of HIF-1α under hypoxic conditions may be a key factor in hypoxia-induced NETosis. Then, we predominantly selected citH3 as a marker to assess NETosis efficiently. The response of dHL-60 cells to hypoxia was indicated by a time-dependent stabilization of HIF-1α, which is sensitive to hypoxic conditions, along with a notable increase in NETosis. These findings suggest that hypoxia may enhance NETosis via the HIF-1α signaling pathway.

Furthermore, we investigated the time-dependent dynamics of NETosis under hypoxic conditions. NETosis reached the highest level observed within our experimental time frame (4 h of hypoxic treatment), comparable to that of the PMA-positive control group. This suggests that hypoxia significantly promoted NETosis through the HIF-1α pathway during this period. In contrast, NETs formation induced by PMA stimulation did not depend on the HIF-1α pathway, suggesting that hypoxia and PMA-induced NETosis differed in mechanism. In summary, hypoxia-induced NETosis was time-dependent, and the stability and expression of HIF-1α under hypoxic conditions may be a key factor driving NETs generation.

### 4.3 Elimination of HIF-1α abrogates hypoxic induction of NETosis

In HIF-1α-deficient mice (22), neutrophils under hypoxic conditions exhibited a suppression of apoptosis in a dose- and time-dependent manner, with a noticeable reduction in cell viability following hypoxic challenge. Based on the observed changes in NETosis dependent on HIF-1α expression, we next aimed to utilize the HIF-1α-targeting inhibitor LW6 to pre-treat dHL-60 cells. LW6 is a novel HIF-1α inhibitor that suppresses HIF-1α expression by promoting its proteasomal degradation without affecting HIF-1β levels (41). This specificity makes LW6 a valuable tool for investigating the role of HIF-1α under hypoxic conditions. This was done to block HIF-1α accumulation prior to exposing the cells to a 4-hour hypoxic challenge in order to assess the formation of NETs and further clarify the role of HIF-1α in hypoxia-induced NETosis.

**Figure 3:**
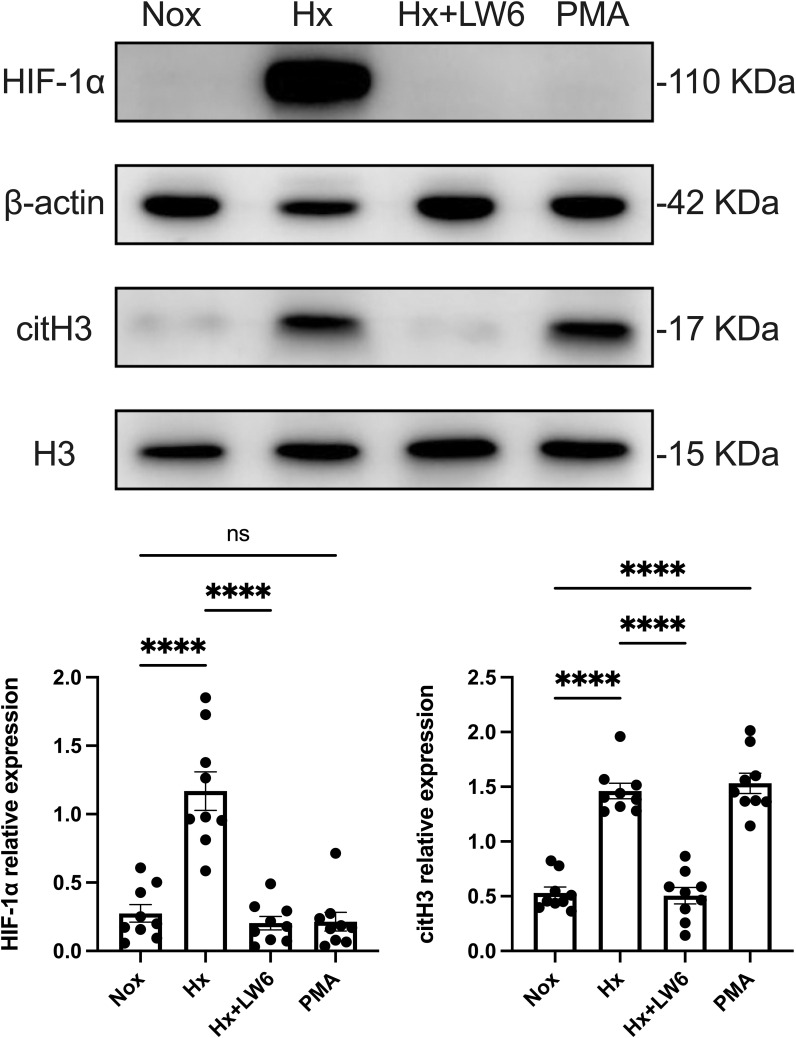
LW6 inhibits HIF-1α expression and reverses hypoxia-induced NETosis. The upper part shows the expression of HIF-1α and citH3 in differentiated dHL−60 cells incubated under hypoxic conditions for 4 h with or without LW6, assessed by Western blot analysis, with β-actin and H3 as loading controls. The lower part shows the quantification of HIF-1α and citH3 protein levels normalized to β-actin or H3, respectively. n = 3 biological replicates, with three technical replicates per sample. ****p < 0.0001, ns, not significant.

Western blot analysis, as shown in **Figure 3**, demonstrated a significant increase in HIF-1α protein expression under hypoxic conditions, accompanied by a corresponding upregulation of the NETs biomarker citH3. However, pre-treatment with LW6 effectively blocked hypoxia-induced HIF-1α expression and significantly reduced citH3 levels. These results suggest that HIF-1α plays a critical role in hypoxia-induced NETosis, and its inhibition can effectively reverse the generation of NETs.

As a positive control, the PMA treatment group showed no significant increase in HIF-1α expression but a substantial rise in citH3 levels. This indicates that while PMA can effectively induce NETs formation in dHL-60 cells, this process is independent of HIF-1α expression.

**Figure 4:**
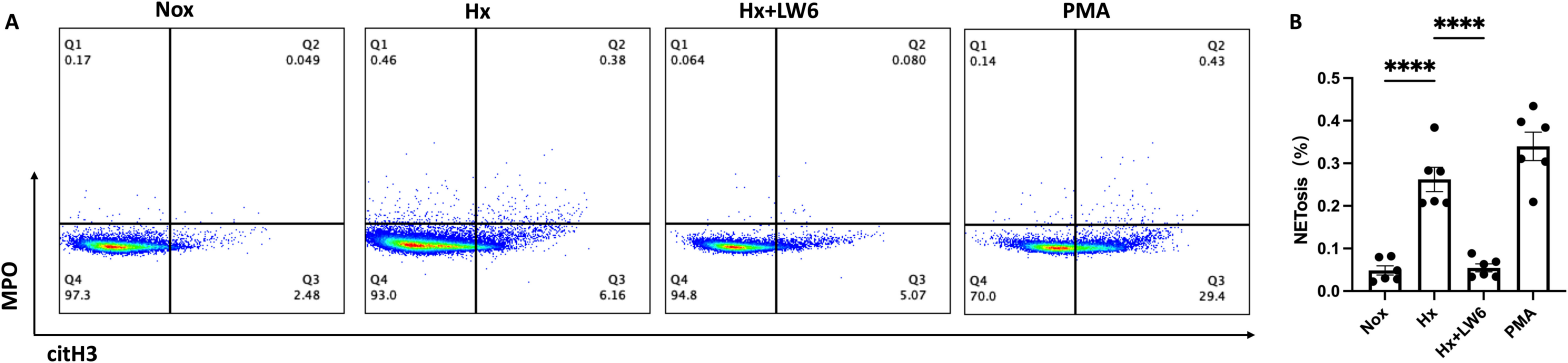
Flow cytometric analysis of LW6 inhibition on hypoxia-induced NETosis. **(A)** Representative FACS plot (n=6) showing the percentage of NETs formation as assessed by flow cytometry in normoxia, hypoxia, hypoxia with LW6, and PMA treated samples and **(B)** its statistical analysis (n=6). ****p < 0.0001.

In addition to the western blot analysis, flow cytometry was used to further validate the promoting effect of hypoxia on NETs formation and the critical role of HIF-1α in this process. The proportion of citH3^+^/MPO^+^ neutrophils was used as an indicator of NETosis. As shown in **Figure 4**, the hypoxia group exhibited a significant increase in NETosis compared to the normoxia group (p < 0.0001), which was consistent with the western blot findings. Furthermore, the HIF-1α inhibitor significantly reduced the proportion of citH3^+^/MPO^+^ neutrophils induced by hypoxia (p < 0.0001), suggesting that HIF-1α plays a pivotal regulatory role in hypoxia-mediated NETosis. Notably, the NET levels following HIF-1α inhibition were similar to those observed in the normoxia group, indicating that the hypoxia-induced NETosis could be effectively reversed by HIF-1α inhibition.

### 4.4 Hypoxia induced NETosis via HIF-1α-dependent rewiring of the glycolytic pathway

HIF-1α plays a crucial role in maintaining cellular energy supply by promoting glycolysis under hypoxic conditions (42) and has been shown to regulate neutrophil metabolism in hypoxic inflammatory environments (20). Based on this, we aimed to determine whether the effects of hypoxia on NETosis are mediated through a HIF-1α-dependent glycolytic pathway.

**Figure 5:**
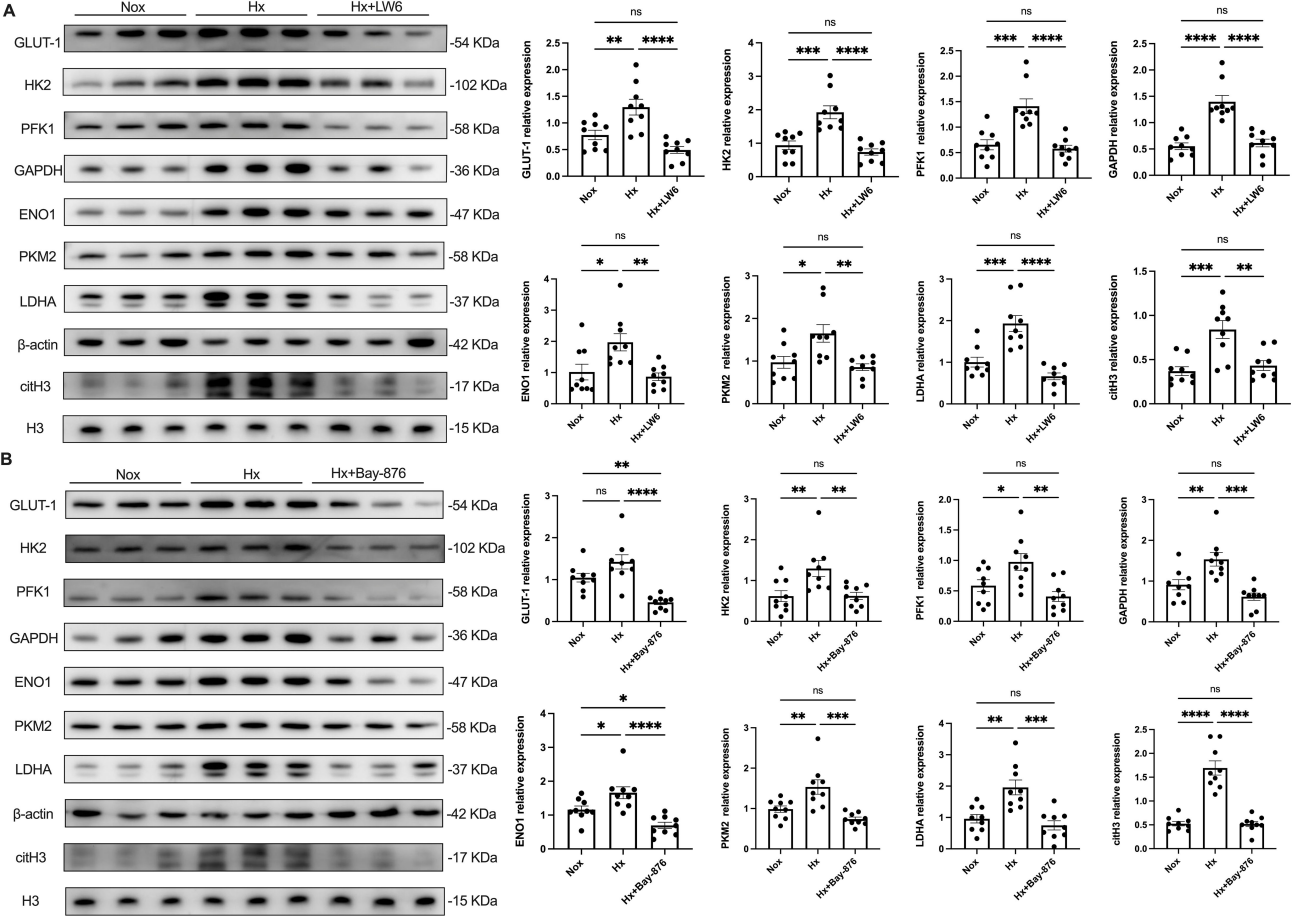
HIF-1α plays a key regulatory role in hypoxia-induced glycolysis and NETosis. dHL−60 were incubated under hypoxic conditions for various times (4 h) with or without LW6 **(A)** or Bay-876 **(B)**, and the expression of GLUT-1, HK2, PFK1, GAPDH, ENO1, PKM2, LDHA, and citH3 were detected by western blot. Expression of glycolysis‐related enzymes and citH3 protein levels were normalized to the β–actin or H3 protein, respectively. n = 3 biological replicates, with three technical replicates per sample. *p < 0.05, **p < 0.01, ***p < 0.001, ****p < 0.0001, ns, not significant.

After 4 h of hypoxic stimulation, dHL-60 cells exhibited a significant upregulation of glycolysis-related enzymes, including GLUT-1, HK2, PFK, GAPDH, ENO1, PKM2, and LDHA, along with an increase in the NETosis marker citH3, as well as elevated expression of PADI4 (**Supplementary Figure S9**), compared to the normoxic control group (**Figure 5**). Given the central role of HIF-1α in hypoxia-induced NETosis, we further investigated the function of HIF-1α-dependent glycolysis in NET formation using the HIF-1α inhibitor LW6. Pre-treatment with LW6 markedly attenuated the expression of glycolytic enzymes, PADI4 and citH3 leading to a significant reduction in NETosis levels under hypoxia.

**Figure 6:**
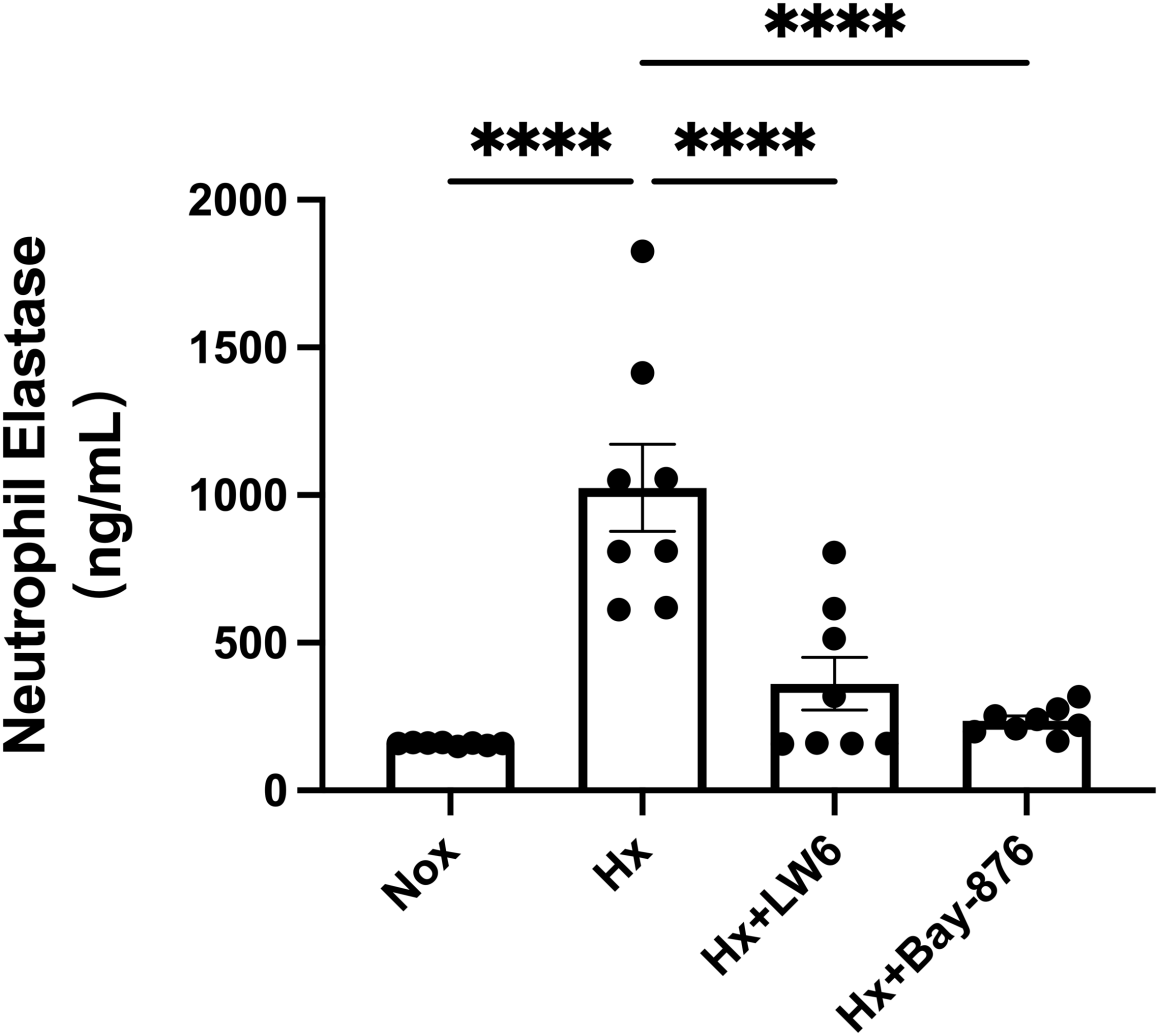
Inhibition of glycolysis by Bay-876 reduces hypoxia-induced neutrophil elastase (NE) release. dHL-60 cells were cultured under normoxic or hypoxic conditions for 4 hours. The level of NE released into the cell culture supernatant was measured by ELISA and statistically analyzed. n = 8. ****p < 0.0001.

**Figure 7:**
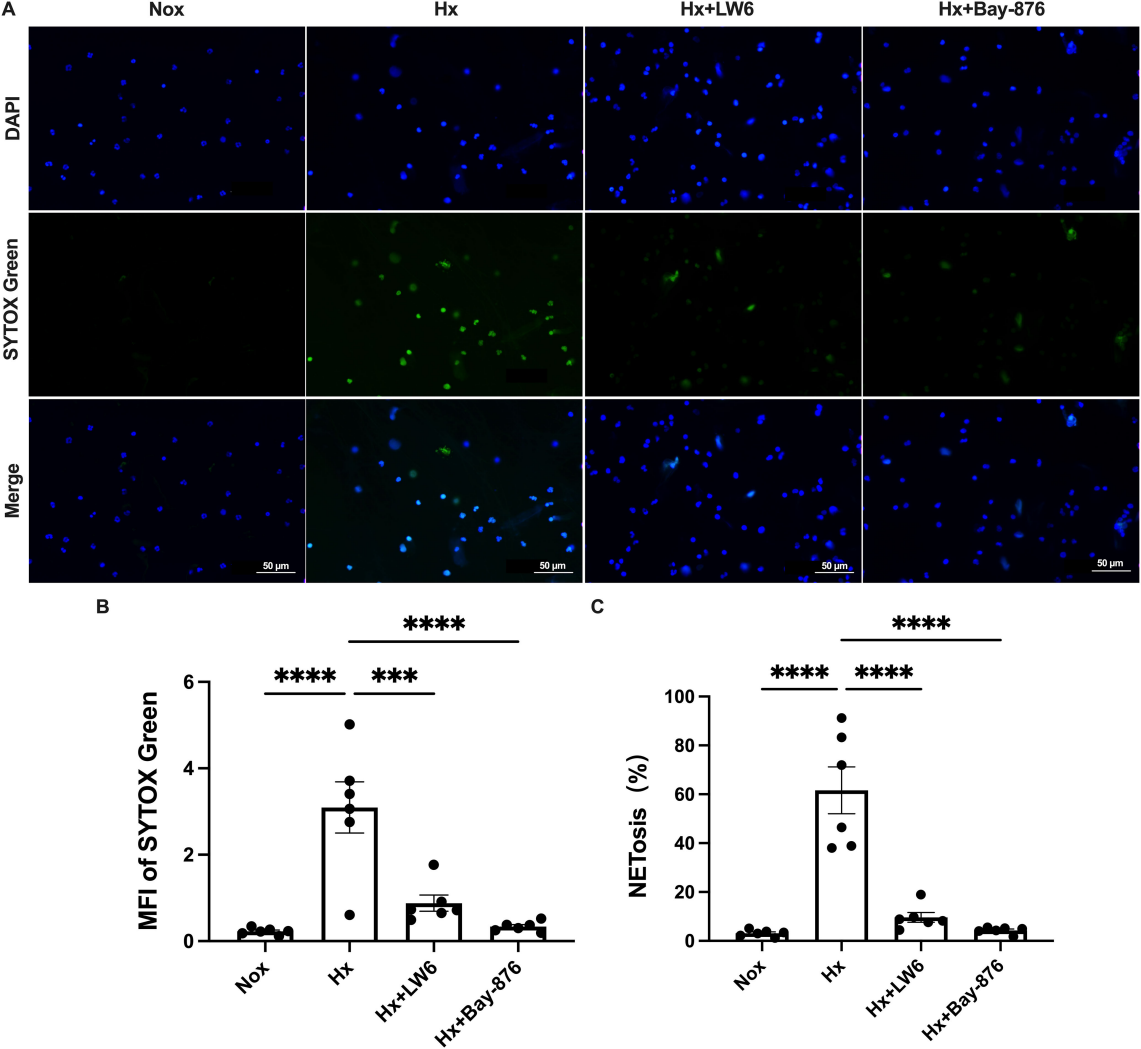
HIF-1α-dependent glycolysis regulates NETosis during hypoxia. **(A)** Representative fluorescence micrographs depicting NET-release of neutrophils. **(B)** Quantification of the mean fluorescence intensity (MFI) of each group of SYTOX Green. **(C)** Percentage of NETosis. n=6. ***p < 0.001, ****p < 0.0001.

To further dissect the role of glycolysis in NETosis, we utilized Bay-876, a GLUT1-specific inhibitor, to suppress glycolytic flux in dHL-60 cells. Western blot analysis revealed that Bay-876 treatment effectively reduced the expression of key glycolytic enzymes, alongside a significant decrease in PADI4 and citH3 levels under hypoxia (**Figure 5**, **Supplementary Figure S9**). Consistently, suppression of glycolysis with Bay-876 led to a notable reduction in NETosis, as evidenced by decreased NE release in the culture supernatant (**Figure 6**) and a significant reduction in both the mean fluorescence intensity (MFI) of SYTOX Green and the percentage of NET-forming neutrophils (**Figure 7**). These findings further confirm that glycolysis is essential for hypoxia-induced NETosis, and inhibition of glycolytic metabolism effectively suppresses this process.

Together, our results demonstrate that hypoxia promotes NETosis through a HIF-1α-dependent glycolytic pathway, and targeting glycolysis via HIF-1α inhibition or GLUT1 blockade can significantly attenuate this response.

## 5 Discussion

In this study, we demonstrate that hypoxia induces persistent NETosis in both human peripheral blood neutrophils from healthy individuals and DMSO-induced dHL-60 cells. We also observed that prolonged hypoxic exposure enhances NETosis in differentiated HL-60 neutrophils. These findings align with previous reports showing that hypoxia contributes to NETosis in conditions such as hypoxic-ischemic brain injury (43), chronic obstructive pulmonary disease (44), and gastric cancer (45), where hypoxic microenvironments not only recruit neutrophils but also trigger NETosis. Our study provides further insights into the characteristics of NETosis under hypoxic conditions, emphasizing the role of HIF-1α-driven glycolytic signaling.

HIF-1α functions as a key oxygen regulator, rapidly accumulating in cells under hypoxia and acting as a critical transcription factor that enhances neutrophil bactericidal activity (22). McInturff et al. demonstrated that cobalt chloride (CoCl_2_), a HIF-1α agonist, induces NETosis via iron chelation (46). In line with this mechanism, our findings support the role of HIF-1α in NET formation, as hypoxia-induced NET release in dHL-60 cells was HIF-1α-dependent, and selective HIF-1α inhibitors reversed this effect.

**Figure 8:**
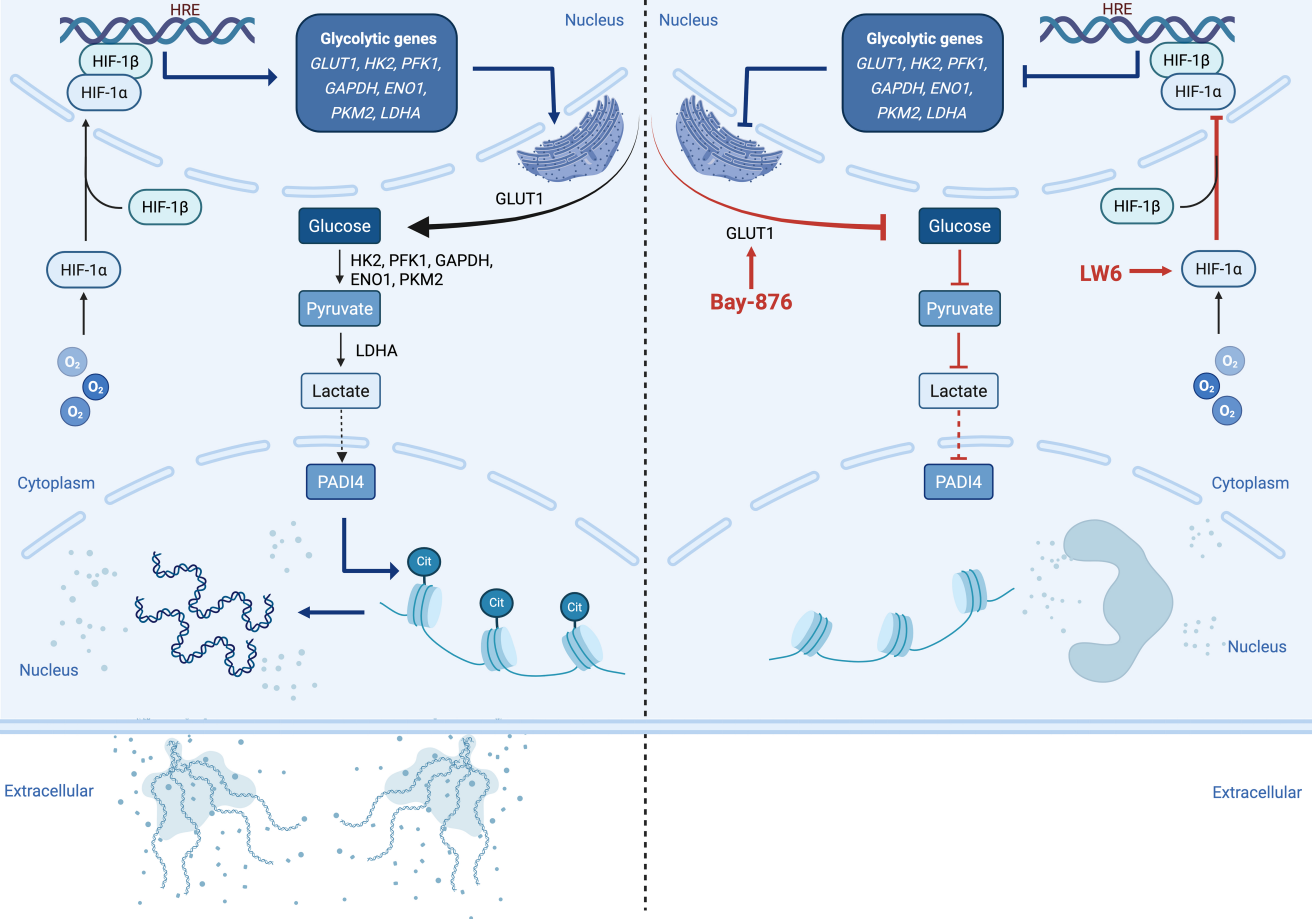
Graphical Abstract. Under hypoxic conditions, HIF-1α is activated in the neutrophil nucleus. This activation promotes the transcription of key glycolytic enzymes, including GLUT1, HK2, PFK1, GAPDH, ENO1, PKM2, and LDHA, thereby enhancing intracellular glycolysis and increasing lactate production. Elevated lactate may activate PADI4, which in turn induces histone citrullination and chromatin decondensation, leading to the release of NETs into the extracellular space (left panel). Treatment with HIF-1α inhibitor LW6 or glycolysis inhibitor Bay-876 blocks this pathway, effectively reversing NETosis under hypoxic conditions (right panel), highlighting the critical role of HIF-1α-dependent glycolysis in NETosis under low oxygen stress.

From a metabolic perspective, neutrophils rely primarily on glycolysis for ATP production due to their limited mitochondria. The HIF-1α-dependent glycolytic program is crucial for supporting neutrophil function. Recent studies indicate that NET formation depends heavily on glucose uptake and glycolytic activity, with both ATP synthase and glycolysis inhibitors suppressing NETosis (47). Additionally, Wei et al. found that 2-DOG, a glycolysis inhibitor, significantly reduced NET formation in neutrophils from acute-on-chronic liver failure patients (48). These findings suggest that metabolic reprogramming underlies neutrophil functional changes and that NET formation imposes a high glycolytic demand, influencing disease progression. Our study further confirms that hypoxia-induced NETosis is strictly dependent on glucose uptake and glycolysis, with HIF-1α playing a central role in this process (**Figure 8**).

While our study identifies HIF-1α-driven glycolysis as a key driver of NETosis, lactate, the final product of glycolysis, may also modulate NETs formation through metabolic signaling or redox modulation. A previous study demonstrated that D-lactic acid can induce NET formation in bovine neutrophils via monocarboxylate transporter 1 (MCT1)-mediated uptake, leading to enhanced neutrophil adhesion and CD11b expression (49). Since PADI4 is a key enzyme mediating histone citrullination during NETosis (50), it is plausible that lactate may influence NETosis by affecting PADI4 activity. Moreover, the enzymatic function of PADI family members is sensitive to changes in cellular metabolic states (51) and pH (52), both of which can be altered by lactate accumulation under hypoxic conditions. Although our data do not directly demonstrate a regulatory effect of lactate on PADI4, this potential interaction merits further investigation. Additionally, although dHL-60 cells are a widely used neutrophil-like model, their energy metabolism and HIF-1α responses may not fully replicate those of primary neutrophils. While prior studies suggest that dHL-60 cells exhibit metabolic features similar to differentiated, mature primary neutrophils (53), further validation in primary neutrophils or *in vivo* models is essential to improve translational relevance.

Moreover, Alfaro et al. demonstrated that IL-8 in the hypoxic tumor microenvironment induces NET extrusion in human myeloid-derived suppressor cells (54). Recently, Tohme et al. reported that NETs promote the development and progression of colorectal liver metastases, highlighting that intratumoral hypoxia exacerbates NET formation in growing metastatic tumors (55). Inspired by these findings, our recent study preliminarily explored NETosis in patients with hypoxia-induced pulmonary hypertension (HPH) and in corresponding animal models. Our unpublished data suggest that circulating NET levels are significantly elevated in HPH patients and positively correlate with disease severity. In HPH mouse models, NETs accumulate in pulmonary tissues and are closely associated with pulmonary vascular remodeling and right ventricular dysfunction. These findings indicate that NETosis is also involved in the progression of hypoxia-related diseases. Therefore, to better understand NETs as a novel pathogenic factor, it is crucial to elucidate their formation mechanisms under specific pathologically relevant oxygen conditions, thereby advancing their potential as therapeutic targets.

In conclusion, our study demonstrates that hypoxia-induced NETosis is strictly dependent on glucose uptake and glycolysis, with HIF-1α serving as a key regulatory factor. While this work provides novel insights, further research is needed to refine the metabolic framework of NETosis, explore its therapeutic potential, and validate its role *in vivo*.

## Data Availability

The original contributions presented in the study are included in the article/**Supplementary Material**. Further inquiries can be directed to the corresponding authors.
