# Supplementary material for: In vitro study: HIF-1α-dependent glycolysis enhances NETosis in hypoxic conditions
Source: Front Immunol. 2025 Apr 28;16:1583587. doi: 10.3389/fimmu.2025.1583587 (PMC12066692; doi:10.3389/fimmu.2025.1583587)
Supplement: Supplementary file 1 [file DataSheet1.docx]

Supplementary Material

# Supplementary Figures


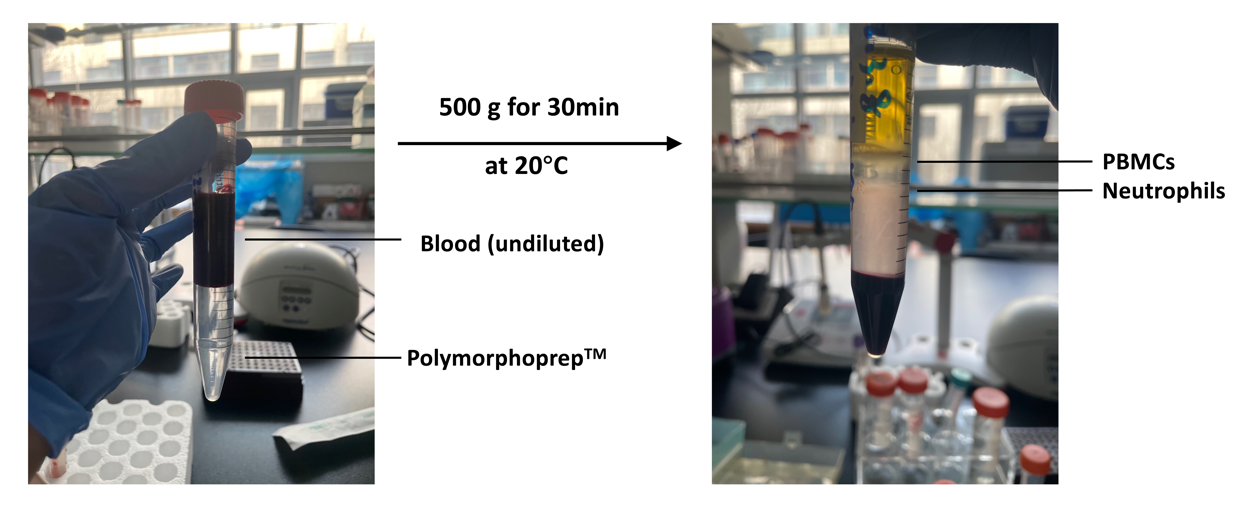


**Supplementary Figure 1. Blood separation with Polymorphprep gradients. (A)** Photograph of the tube filled with the diluted blood sample. **(B)** Photograph of a successful separation post-centrifugation.


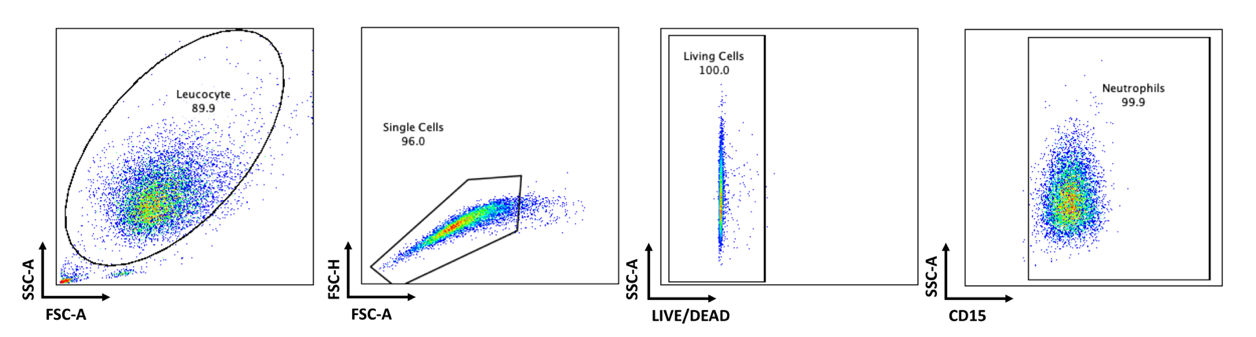


**Supplementary Figure 2. Representative FACS plot of isolated neutrophils from whole blood of healthy donors. (A)** The initial gating was used for peripheral blood cells collected from the Polymorphprep gradient medium using FSC/SSC. **(B)** Single cells were subsequently selected in a FSC-A vs FSC-H dot plot. As shown, neutrophils collected from the medium are >99% viable **(C)** and >99% pure**(D)**. Data are from 6 independent experiments. CD: Cluster of Differentiation; FSC: Forward Scatter; SSC: Side Scatter.


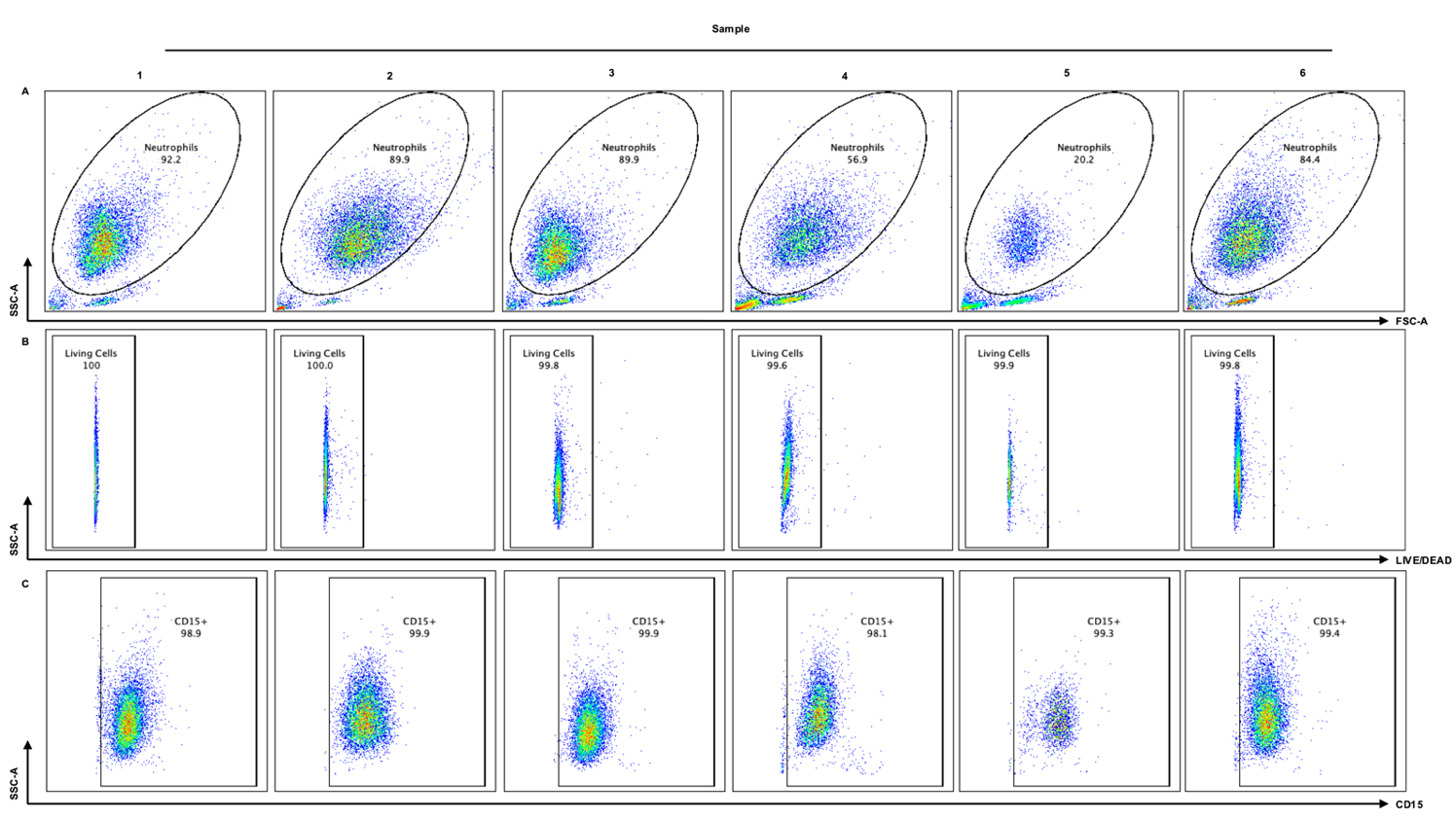


**Supplementary Figure 3.** **Results of flow cytometry identification of peripheral blood neutrophils in 6 samples are shown.** The cells in the combined FSC-SSC profiles showed larger cell volume and larger granularity, a feature consistent with typical neutrophils in flow cytometry physicograms, which initially demonstrated that the isolated cells were highly purified neutrophils.


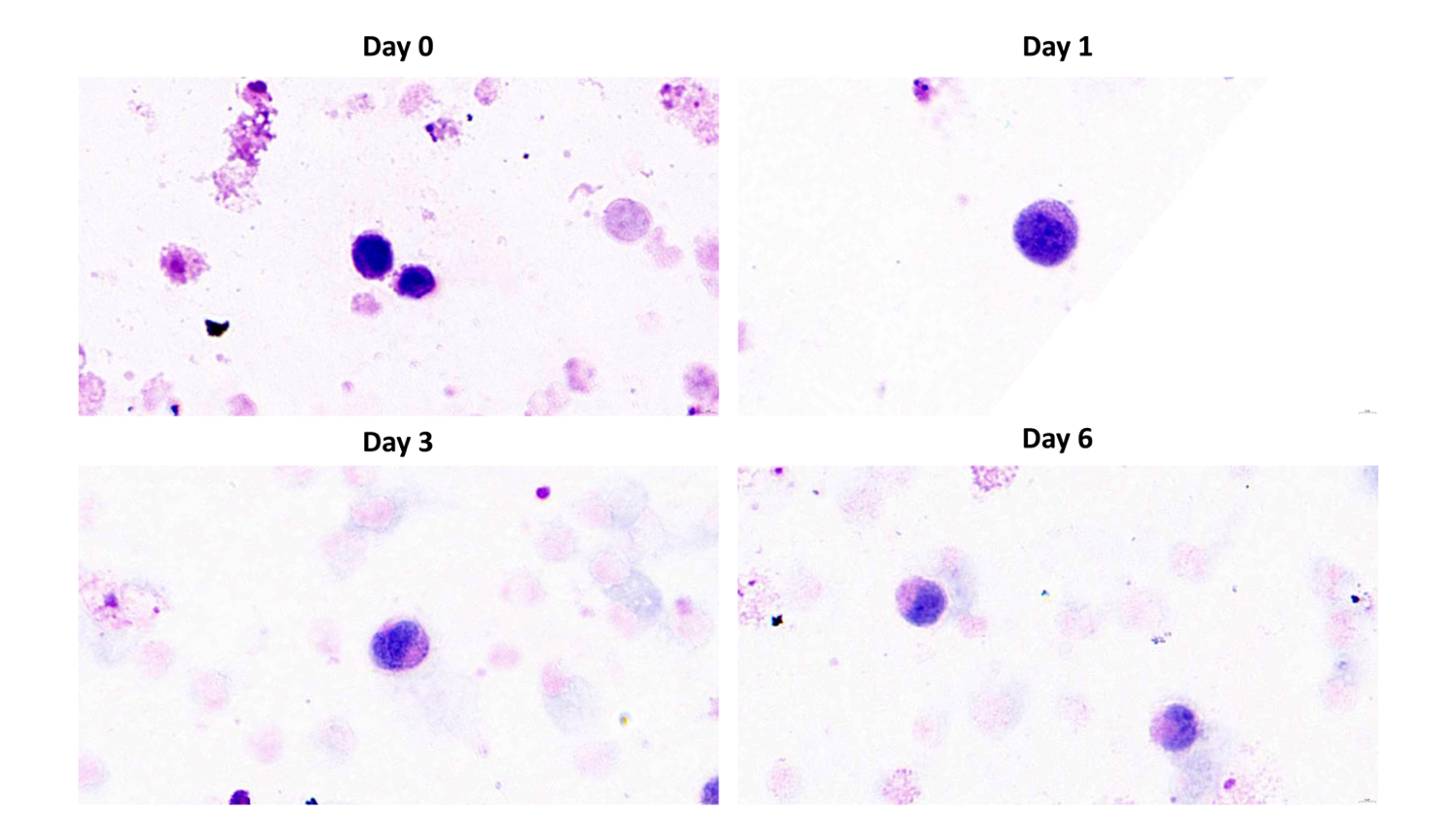


**Supplementary Figure 4.** **Giemsa staining showing the differentiation of HL-60 cells after DMSO induction.** Representative images are shown, with a scale bar of 5 μm.


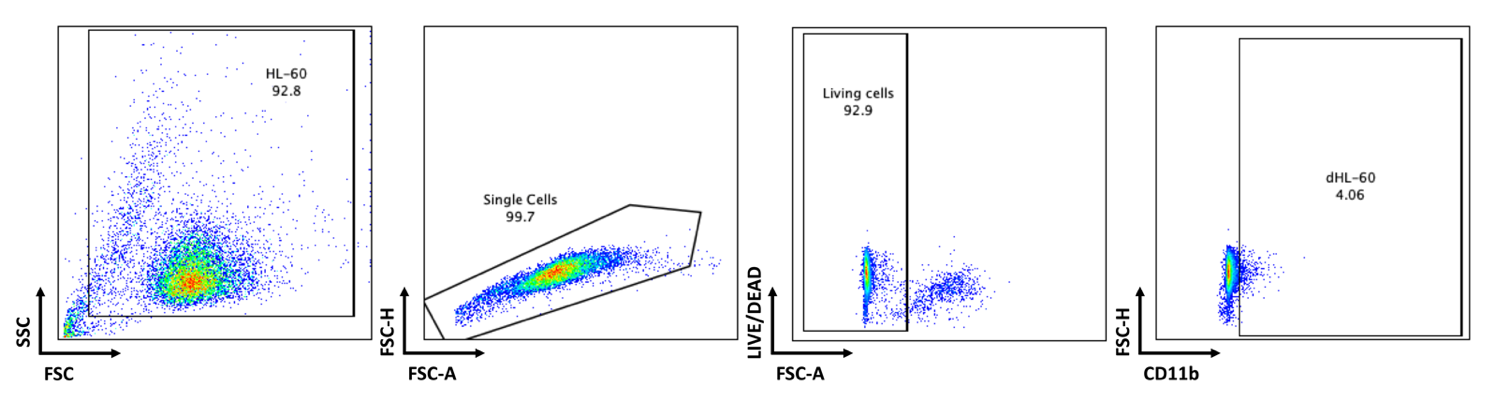


**Supplementary Figure 5.** **Gating strategy for flow cytometric identification of dHL-60 cell viability and purity.** In the FSC-SSC scatter plot, the cell population with relatively larger size and granularity corresponds to HL-60 cells (P1). A combined FSC-A vs. FSC-H plot was used to exclude doublets and select singlet cells (P2) to ensure accurate cell counting. Live neutrophil populations were further identified by excluding Zombie Aqua™-positive dead cells detected in the BV510 channel. Neutrophil differentiation was confirmed by the presence of FITC-conjugated CD11b-positive cells.


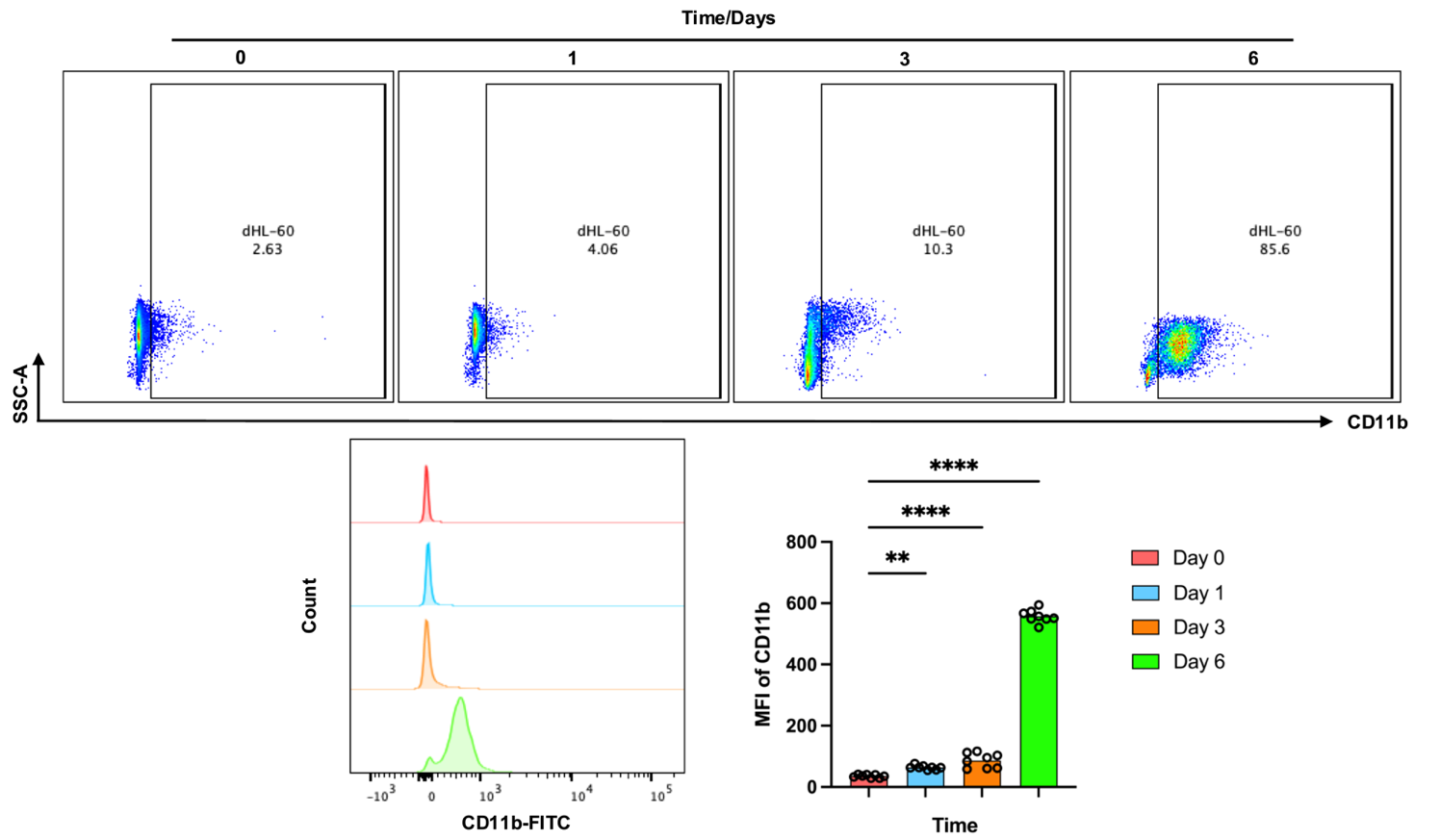


**Supplementary Figure 6.** **Flow cytometric analysis of CD11b expression in HL-60 cells after DMSO induction.** (A) Proportion of CD11b-positive cell populations. (B) Mean fluorescence intensity (MFI) of CD11b and its statistical analysis.


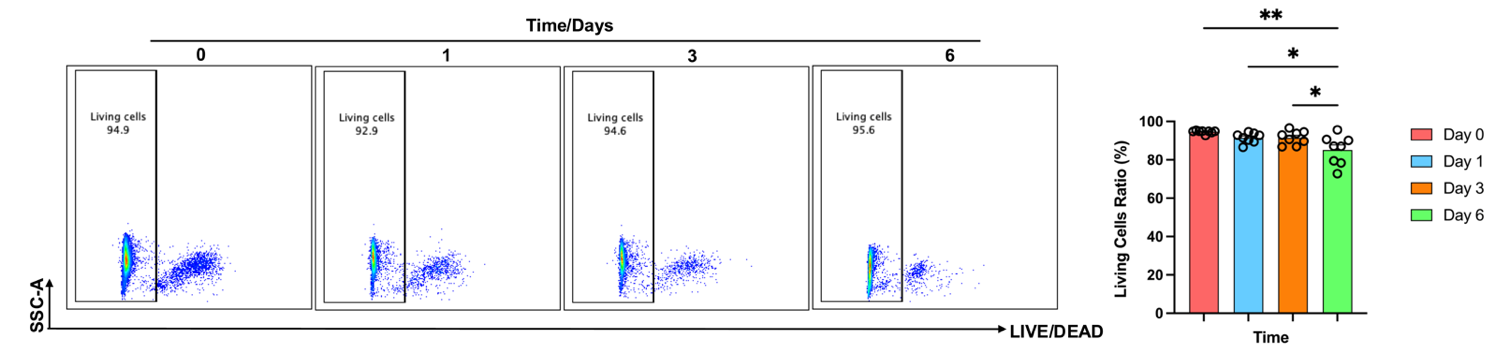


**Supplementary Figure 7. Flow cytometric analysis of HL-60 cell viability after DMSO induction and its statistical analysis.**


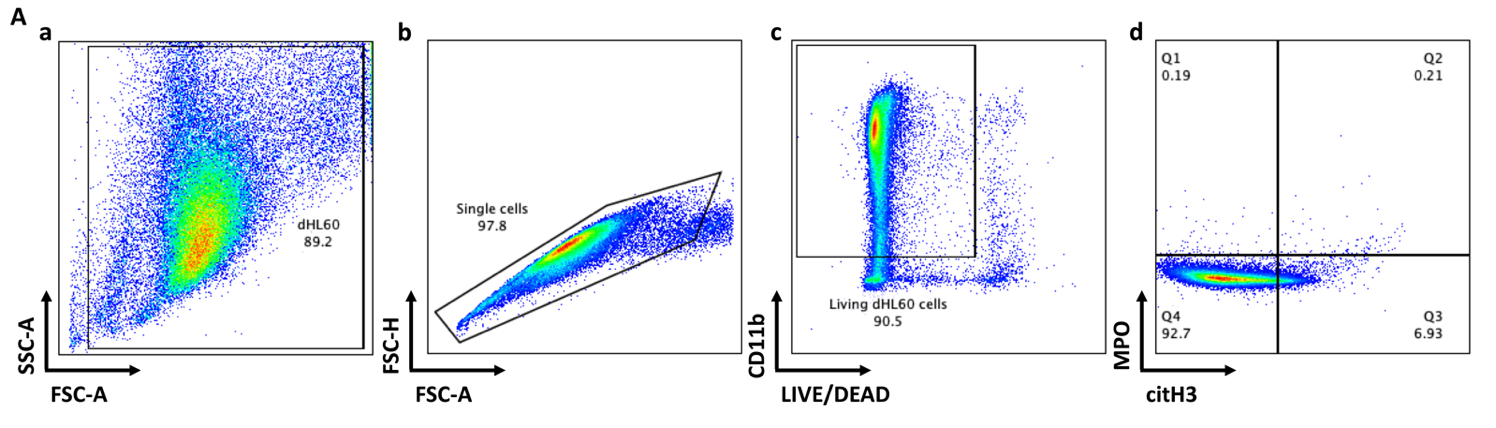


**Supplementary Figure 8. Gating strategy for flow cytometric analysis of NETosis in dHL-60 cells.** First, neutrophils**(a)** were selected from a forward scatter-area vs side scatter dot plot, and single cells**(b)** were subsequently selected in a FSC-A vs FSC-H dot plot. Then, **the neutrophil population(c) was identified by CD11b+, and** dead cells were excluded with Zombie Aqua Fixable Viability Kit. NETosis**(d)** detected as H3cit and MPO double positive cells above threshold (upper right quadrant Q2).


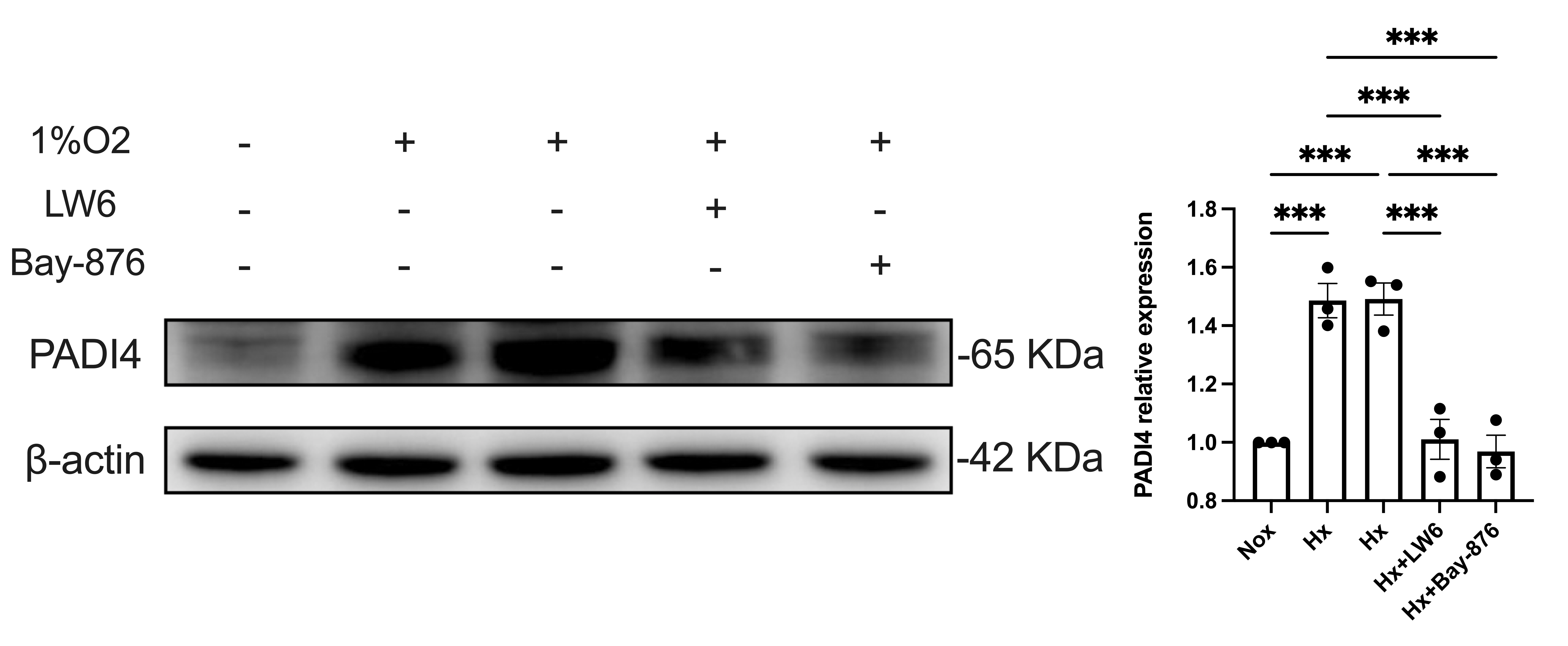


**Supplementary Figure 9. NETosis during hypoxia is caused by increased expression of PADI4.** Western blot analysis of PADI4 expression in dHL-60 cells under normoxic (Nox), hypoxic (Hx), and hypoxic conditions treated with LW6 or Bay-876. β-actin served as a loading control. n = 3.
